# The effects of salbutamol on epithelial ion channels depend on the etiology of acute respiratory distress syndrome but not the route of administration

**DOI:** 10.1186/1465-9921-15-56

**Published:** 2014-05-02

**Authors:** Christopher Uhlig, Pedro L Silva, Débora Ornellas, Raquel S Santos, Paulo J Miranda, Peter M Spieth, Thomas Kiss, Michael Kasper, Bärbel Wiedemann, Thea Koch, Marcelo M Morales, Paolo Pelosi, Marcelo Gama de Abreu, Patricia RM Rocco

**Affiliations:** 1 Laboratory of Pulmonary Investigation, Carlos Chagas Filho Biophysics Institute, Federal University of Rio de Janeiro, Av. Carlos Chagas Filho s/n, Bloco G-014, Rio de Janeiro, RJ 21941-902, Brazil; 2 Department of Anesthesiology and Intensive Care Therapy, Pulmonary Engineering Group, University Hospital Dresden, Technische Universität Dresden, Fetscherstr, Dresden 74, 01307, Germany; 3 Laboratory of Cellular and Molecular Physiology, Carlos Chagas Filho Biophysics Institute, Federal University of Rio de Janeiro, Av. Carlos Chagas Filho s/n, Bloco G2-048, Ilha do Fundão, Rio de Janeiro, RJ 21941-902, Brazil; 4 Institute of Anatomy, Faculty of Medicine, Technische Universität Dresden Fetscherstr, Dresden 74, 01307, Germany; 5 Institute of Biometrics and Medical Informatics, Faculty of Medicine, Technische Universität Dresden, Dresden, Fetscherstr, 74 Dresden 01307, Germany; 6 IRCCS AOU San Martino-IST, Department of Surgical Sciences and Integrated Diagnostics, University of Genoa, Largo Rosanna Benzi 8, 16132, Genoa, Italy

**Keywords:** Salbutamol, Acute respiratory distress syndrome, Elastance, Alveolar epithelial cells, Epithelial sodium channel, Edema

## Abstract

**Introduction:**

We investigated the effects of intravenous and intratracheal administration of salbutamol on lung morphology and function, expression of ion channels, aquaporin, and markers of inflammation, apoptosis, and alveolar epithelial/endothelial cell damage in experimental pulmonary (p) and extrapulmonary (exp) mild acute respiratory distress syndrome (ARDS).

**Methods:**

In this prospective randomized controlled experimental study, 56 male Wistar rats were randomly assigned to mild ARDS induced by either intratracheal (n = 28, ARDSp) or intraperitoneal (n = 28, ARDSexp) administration of *E. coli* lipopolysaccharide. Four animals with no lung injury served as controls (NI). After 24 hours, animals were anesthetized, mechanically ventilated in pressure-controlled mode with low tidal volume (6 mL/kg), and randomly assigned to receive salbutamol (SALB) or saline 0.9% (CTRL), intravenously (i.v., 10 μg/kg/h) or intratracheally (bolus, 25 μg). Salbutamol doses were targeted at an increase of ≈ 20% in heart rate. Hemodynamics, lung mechanics, and arterial blood gases were measured before and after (at 30 and 60 min) salbutamol administration. At the end of the experiment, lungs were extracted for analysis of lung histology and molecular biology analysis. Values are expressed as mean ± standard deviation, and fold changes relative to NI, CTRL *vs.* SALB.

**Results:**

The gene expression of ion channels and aquaporin was increased in mild ARDSp, but not ARDSexp. In ARDSp, intravenous salbutamol resulted in higher gene expression of alveolar epithelial sodium channel (0.20 ± 0.07 *vs.* 0.68 ± 0.24, p < 0.001), aquaporin-1 (0.44 ± 0.09 *vs.* 0.96 ± 0.12, p < 0.001) aquaporin-3 (0.31 ± 0.12 *vs.* 0.93 ± 0.20, p < 0.001), and Na-K-ATPase-α (0.39 ± 0.08 *vs.* 0.92 ± 0.12, p < 0.001), whereas intratracheal salbutamol increased the gene expression of aquaporin-1 (0.46 ± 0.11 *vs*. 0.92 ± 0.06, p < 0.001) and Na-K-ATPase-α (0.32 ± 0.07 *vs.* 0.58 ± 0.15, p < 0.001). In ARDSexp, the gene expression of ion channels and aquaporin was not influenced by salbutamol. Morphological and functional variables and edema formation were not affected by salbutamol in any of the ARDS groups, regardless of the route of administration.

**Conclusion:**

Salbutamol administration increased the expression of alveolar epithelial ion channels and aquaporin in mild ARDSp, but not ARDSexp, with no effects on lung morphology and function or edema formation. These results may contribute to explain the negative effects of β2-agonists on clinical outcome in ARDS.

## Introduction

Acute respiratory distress syndrome (ARDS) is characterized by an increased permeability of the alveolar-capillary membrane [1]. In addition, fluid clearance is reduced, resulting in congestion, atelectasis and alveolar edema, which can impair gas exchange and respiratory mechanics [2], and even increase mortality [3]. Lung fluid homeostasis is controlled by different mechanisms [4]. The removal of alveolar edema depends on the transport of salt and water across the alveolar epithelial sodium channels (ENaC), followed by extrusion of fluid into the lung interstitium via basolaterally located Na-K-ATPase. The resulting gradient in Na^+^ concentration absorbs water from the airspace by a transcellular route via aquaporins (AQP). Moreover, increased apoptosis and necrosis of alveolar epithelial cells result in a loss of barrier and transport properties [5].

The potential of β2-adrenoreceptor agonists to reduce alveolar-capillary permeability and increase fluid clearance has been investigated [6-8]. These drugs upregulate apical Na^+^ and Cl^−^ channels in alveolar type II cells, reduce neutrophil influx into the lungs, and inhibit inflammatory cytokines [9-11]. Different experimental studies have shown that nebulized salbutamol reduces lung edema [12,13]. In a single-center phase II trial [14], intravenous β2-agonist administration decreased lung edema in ARDS patients. However, in multicenter randomized controlled trials, intravenous [15] or nebulized [16] administration of β2-agonists increased mortality or failed to show beneficial effects, respectively, in ARDS patients. Nevertheless, those studies [14-16] included patients with ARDS of different etiologies and used different routes of administration, which could have affected the results. Yet, to our knowledge, these factors have not been systematically addressed. Since in pulmonary (p) ARDS the primary damage occurs in alveolar epithelial cells [17-19], the effects of salbutamol on ion channel gene expression would be more pronounced. In contrast, in extrapulmonary (exp) ARDS, the primary damage occurs in endothelial cells and in this case salbutamol would be expected to have less effect on ion channel gene expression regardless of the route of administration.

The aim of this study was to investigate the effects of salbutamol administered intravenously (i.v.) and intratracheally (i.t.) in mechanically ventilated rats with mild ARDSp and ARDSexp on: 1) lung function and histology, and 2) biological markers associated with alveolar fluid clearance, inflammation, apoptosis, and damage inflicted on alveolar epithelial and endothelial cells.

## Material and methods

This study was approved by the Ethics Committee of the Carlos Chagas Filho Institute of Biophysics, Health Sciences Centre, Federal University of Rio de Janeiro, Brazil. All animals received humane care in compliance with the “Principles of Laboratory Animal Care” formulated by the National Society for Medical Research and the “Guide for the Care and Use of Laboratory Animals” prepared by the National Academy of Sciences, USA.

### Induction of ARDS and anesthesia

Sixty adult male Wistar rats (242–336 g) were used. ARDSp (n = 28) was induced by intratracheal injection of *E. coli* lipopolysaccharide (LPS, serotype 055:B5; Sigma Aldrich, São Paulo, SP, Brazil) (200 μg suspended in 100 μL saline 0.9%), and ARDSexp (n = 28) by intraperitoneal injection of LPS (1000 μg suspended in 1000 μL saline 0.9%). For this purpose, rats were anesthetized with sevoflurane (2.5 vol.%; Cristália, São Paulo, SP, Brazil). After recovering from anesthesia, all rats were kept under observation in cages. These doses of LPS were chosen because they can yield a 1.5-fold-increase in static lung elastance in both ARDSp and ARDSexp, according to a previous study of our group [18]. Four rats, which did not receive LPS or mechanical ventilation, served as non-injured controls for molecular biology analysis.

Twenty-four hours after ARDS induction, rats were premedicated intraperitoneally (i.p.) with 1–2 mg/kg midazolam (Dormicum; União Química, São Paulo, SP, Brazil) and 50–100 mg/kg ketamine (Vetanarcol; König Laboratories Brazil, Santana de Parnalha, SP, Brazil). An intravenous catheter (Jelco 24G) was inserted into the tail vein for continuous infusion of 2 mg/kg/h midazolam, 100 mg/kg/h ketamine, and 7 mL/kg/h Ringer’s lactate (B. Braun, Crissier, Switzerland). Animals were kept in the supine position throughout the experiment.

### Preparation and instrumentation

Animals were tracheotomized and a polyethylene catheter (PE-50) was introduced into the right internal carotid artery for blood sampling and mean arterial blood pressure (MAP) measurement. Electrocardiogram (ECG), MAP and rectal temperature were continuously recorded (Networked Multiparameter Veterinary Monitor LifeWindow 6000 V, Digicare Animal Health, Florida, USA). Body temperature was maintained at 38.5°C ± 1°C using a heating pad (Insight Ltda, São Paulo, SP, Brazil). The left jugular vein was cannulated (Jelco® 24G catheter, Johnson & Johnson, São José dos Campos, Brazil) for infusion of salbutamol or Ringer’s lactate.

### Measurements and experimental protocol

After the end of preparation, arterial blood gases (iSTAT System, CG8+ cartridge; Abbott Point of Care Inc., Princeton, NJ, USA) and hemodynamics were measured (Baseline 1 – BL1). Animals were paralyzed (pancuronium bromide, 2 mg/kg i.v.) and mechanically ventilated (Servo-i, MAQUET, Solna, Sweden) in pressure-controlled mode with tidal volume (V_T_) = 6 mL/kg, respiratory rate (RR) = 80 breaths/min, inspiratory-to-expiratory ratio (I:E) = 1:2, fraction of inspired oxygen (FIO_2_) = 0.4, and positive end-expiratory pressure (PEEP) = 3 cmH_2_O. Gelafundin® (B. Braun, Melsungen, Germany) was administered (in steps of 0.5 mL) to maintain MAP >60 mmHg. After 5 min of stabilization, respiratory system mechanics, arterial blood gases and hemodynamics were measured (Baseline 2 – BL2).

**Figure 1:**
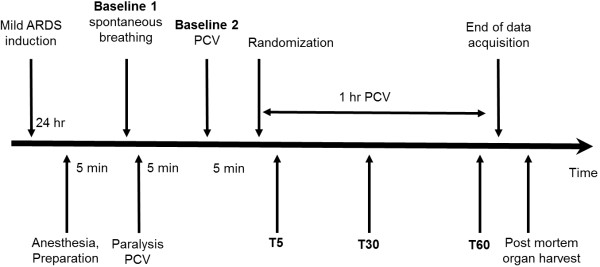
**Timeline representation of the experimental protocol.** ARDS = acute respiratory distress syndrome; T5, T30, and T60 = measurement points at 5, 30, and 60 min after initiation of intervention; PCV = pressure-controlled ventilation.

In both ARDSp and ARDSexp groups, rats were then randomly assigned to one of four subgroups (n = 7/subgroup) to receive i) intravenous salbutamol (SALB-i.v., 10 μg/kg/hour for 30 min) (Hipolabor, Sabará, MG, Brazil), ii) intravenous saline 0.9% (CTRL-i.v., 3 mL for 30 min), iii) intratracheal salbutamol (SALB-i.t., 25 μg suspended in 50 μL), or iv) intratracheal saline 0.9% (CTRL-i.t., 50 μL). Intratracheal applications were performed using a high pressure syringe (Model FMJ-250; Penn-Century, Inc., Wyndmoor, PA, USA) connected to an aerosolizer device (Microsprayer Model IA-1C; Penn-Century, Inc., Wyndmoor, PA, USA). Intratracheal β2-agonist doses were chosen based on a previous report [12] and pilot experiments showing hemodynamic instability when heart rate (HR) increased more than 20%. Intravenous doses were selected based on pilot experiments to achieve an increase of ≈ 20% in HR. After 1-hour mechanical ventilation, a laparotomy was performed and heparin (1000 IU; Hipolabor, Sabará, MG, Brazil) was injected i.v.. Animals were then killed with thiopental (25 mg i.v.; Cristália, São Paulo, SP, Brazil), and their lungs extracted for histological and molecular biology analysis (Figure 1).

### Hemodynamics

HR and MAP were measured at BL1 and BL2, and also at 5 min (T5), 30 min (T30) and 60 min (T60) during the experimental period. T5 measurements were performed to assess the immediate hemodynamic effects of salbutamol.

### Blood gases and respiratory system mechanics

Arterial blood gases were measured at BL1, BL2, T30, and T60. Airway pressure (Paw) and airflow were measured with a differential pressure transducer (UT-PDP-300; SCIREQ, Montreal, Quebec, Canada) and continuously recorded. All signals were filtered (100 Hz), amplified in a 4-channel signal conditioner (SC-24; SCIREQ, Montreal, Quebec, Canada), and sampled at 200 Hz with a 12-bit analogue-to-digital converter (NI-DAQmx 8.7.1, Austin, Texas, USA). Peak (Paw_peak_) and mean (Paw_mean_) airway pressures were computed. The elastance (E_rs_) and resistance (R_rs_) of the respiratory system were calculated using the equation of motion. Respiratory variables were computed from continuous recordings of airflow and Paw at BL2, T30, and T60 using routines written in MATLAB (version 7.14; The Mathworks Inc., Natick, MA, USA).

### Postmortem processing

For postmortem processing, the trachea was clamped at end-expiration at PEEP of 3 cmH_2_O.

### Wet-to-dry ratio

The wet-to-dry (W/D) ratio was determined in the right middle lobe as described elsewhere [20]. Briefly, the right middle lobe was removed, weighted (wet weight), and then dried in a microwave oven at low power (200 W) for 5 min. The drying process was repeated until the difference between two consecutive lung weight measurements was less than 0.002 g. The last weight recorded represented the dry weight.

### Histology

The right lower lung lobe was fixed in 4% buffered formaldehyde, embedded in paraffin, cut into slices of 3 μm thickness, and stained with hematoxylin-eosin for histological analysis. Photomicrographs at magnifications of ×25, ×100 and ×400 were obtained from eight non-overlapping fields of view per section using a light microscope. Diffuse alveolar damage (DAD) was quantified using a weighted scoring system, as described elsewhere [21]. Briefly, values from 0 to 4 were used to represent the severity of edema, hemorrhage, inflammatory infiltration, and overdistension, with 0 standing for no effect and 4 for maximum severity. Additionally, the extent of each score characteristic per field of view was determined by using values of 0 to 4, with 0 standing for no appearance and 4 for complete involvement. Scores were calculated as the product of severity and extent of each feature, in the range of 0 to 64. Scoring was assessed by an expert in lung pathology (MK) blinded to group assignment.

### Biological markers of inflammation, alveolar epithelial and endothelial cell injury, apoptosis, and alveolar fluid clearance

Quantitative real-time reverse transcription polymerase chain reaction (PCR) was performed to measure the mRNA expression of biological markers associated with inflammation [interleukin (IL)-6, macrophage inflammatory protein (MIP)-2, and tumor necrosis factor (TNF)-α], apoptosis [pro-caspase-3, BH3 interacting-domain death agonist (Bid), and Bcl-2-associated X protein (Bax)], alveolar epithelial type I cell damage [receptor for advanced glycation end-products (RAGE)], endothelial cell injury [vascular cell adhesion molecule (VCAM)-1], and alveolar fluid clearance [ENaC-α, Na-K-ATPase (α subunit), and AQP-1 and −3]. Central slices of the left lung were cut, collected in cryotubes, quick-frozen by immersion in liquid nitrogen, and stored at −80°C. Total RNA was extracted using the SV Total RNA Isolation System (Promega, Fitchburg, WI, USA). RNA concentration was measured by spectrophotometry in Nanodrop® ND-1000. First-strand cDNA was synthesized from total RNA using M-MLV Reverse Transcriptase Kit (Invitrogen, Carlsbad, CA, USA). The primers and Real-Time PCR protocols are described in detail in the Additional file 1.

### Statistical analysis

Values are expressed as mean ± standard deviation (SD) unless otherwise specified. Differences between groups at BL1 and BL2, as well as in gene expression, were tested using one-way ANOVA with Bonferroni correction. Between- and within-groups differences (T5, T30, T60; time, group, and time × group effects) were tested with two-way ANOVA and adjusted for repeated measures according to the Bonferroni method. A mixed linear model followed by Tukey-Kramer test was used to analyze the histological features of the DAD score. All data were tested separately for ARDSp and ARDSexp groups. All statistical analyses were performed using SPSS (version 20; SPSS Inc., Chicago, IL, USA), GraphPad Prism (version 5.01; GraphPad Software, La Jolla, CA, USA) and the mixed procedure of SAS (version 9.2; SAS Institute, Cary, NC, USA). Statistical significance was accepted at α = 0.05.

## Results

The groups did not differ in body weight, dose of anesthesia, amount of crystalloids, and fluid therapy (Additional file 2: Table S2).

**Figure 2:**
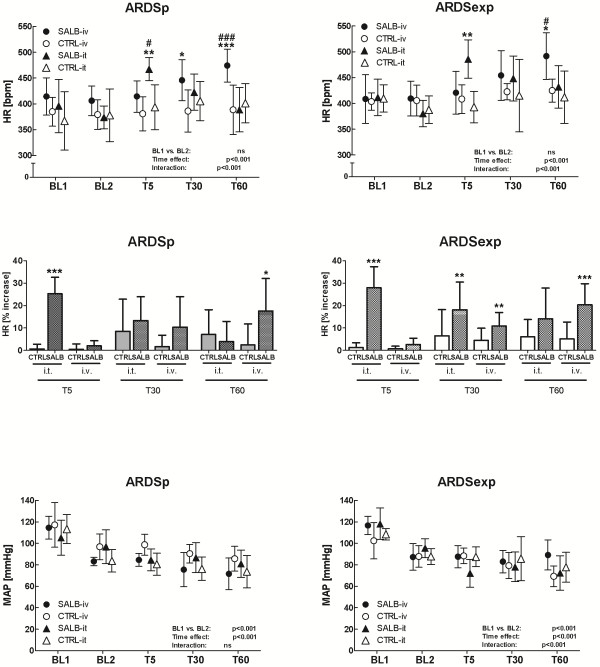
**Heart rate (HR) and mean arterial pressure (MAP) during the experimental protocol.** All values are expressed as mean and standard deviation. Effects of initiation of mechanical ventilation were tested with a paired *t*-test. Differences between and within groups (*group and time effects, T5, T30, T60 as well as their interactions*) were tested separately for ARDSp and ARDSexp using two-way ANOVA and adjusted for repeated measures according to the Bonferroni method. SALB = salbutamol; CTRL = control; i.t. = intratracheally; i.v. = intravenously; BL1 = baseline 1; BL2 = baseline 2; T5, T30, and T60 = 5, 30, and 60 min after initiation of therapy; ARDSp = pulmonary acute respiratory distress syndrome; ARDSexp = extrapulmonary acute respiratory distress syndrome; ns = non-significant; * = p < 0.05 vs. CTRL; ** = p < 0.01 vs. CTRL; *** = p < 0.001 vs. CTRL; # = p < 0.05 vs. SALB; ### = p < 0.001 vs. SALB.

In both ARDSp and ARDSexp, doses of salbutamol were 1.5 ± 0.1 μg/kg/h (i.v.) and 25 ± 0 μg/kg/h (i.t.), leading to an increase in HR with a progressive decrease in MAP, as compared to BL2, regardless of the route of drug administration (Figure 2).

Paw_peak_, E_rs_ and R_rs_ increased over time with the administration of saline or salbutamol in both ARDS groups, regardless of the route of administration (Additional file 2: Table S3).

**Figure 3:**
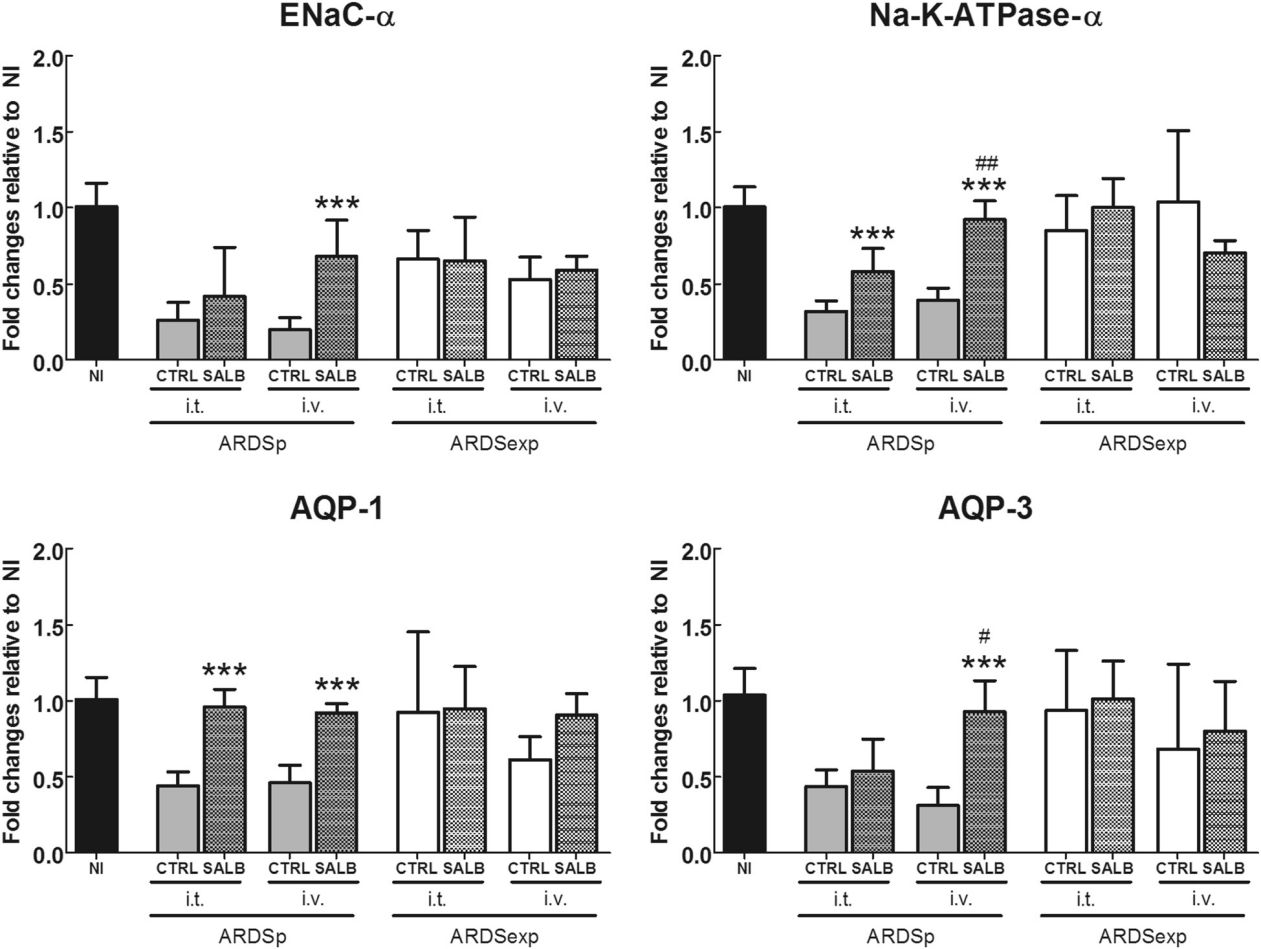
**Gene expression of channels associated with alveolar fluid clearance and pulmonary edema formation.** ENaC-α = alpha subunit of the epithelial sodium channel; Na-K-ATPase-α = alpha subunit of the Na-K-ATPase; AQP-1 = aquaporin 1; AQP-3 = aquaporin 3. Differences between groups (SALB vs. CTRL; SALB-iv vs. SALB-it) were tested separately for ARDSp and ARDSexp using one-way ANOVA followed by Bonferroni correction. Statistical significance was accepted at α = 0.05. All values are expressed as mean and standard deviation. ** = p < 0.01; *** = p < 0.001 vs. CTRL; # = p < 0.05; ## = p < 0.01 vs. SALB-i.t.; NI = non-injured controls; CTRL = control; SALB = salbutamol; i.t. = intratracheally; i.v. = intravenously; ARDSp = pulmonary acute respiratory distress syndrome; ARDSexp = extrapulmonary acute respiratory distress syndrome.

Intravenous injection of salbutamol increased the expression of ENaC-α, AQP-1, AQP-3 and Na-K-ATPase-α in ARDSp, but not in ARDSexp (Figure 3). Intratracheal salbutamol increased the gene expression of AQP-1 and Na-K-ATPase-α in ARDSp. In addition, in ARDSp, the intravenous injection of salbutamol induced higher gene expression of AQP-3 and Na-K-ATPase-α than intratracheal instillation of salbutamol.

In both salbutamol groups, partial pressure of arterial carbon dioxide (PaCO_2_) and arterial pH (pHa) deteriorated over time, irrespective of the etiology of lung injury, while arterial oxygen partial pressure (PaO_2_) showed a greater decrease in ARDSp (Table 1).

**Table 1:** Arterial blood gases

**Variable**	**ARDSp**				**ARDSexp**				
	**CTRL-it**	**SALB-it**	**CTRL-iv**	**SALB-iv**		**CTRL-it**	**SALB-it**	**CTRL-iv**	**SALB-iv**
**PaO**_**2**_**[mmHg (kPa)]**									
BL1	236 ± 61	238 ± 66	227 ± 50	200 ± 46	249 ± 36	249 ± 50	241 ± 42	244 ± 51	
	(31.5 ± 8.0)	(31.8 ± 8.8)	(30.3 ± 6.6)	(26.7 ± 6.1)	(33.2 ± 4.9)	(33.2 ± 6.6)	(32.2 ± 5.6)	(32.5 ± 6.8)	
BL2	230 ± 88	249 ± 65	238 ± 74	246 ± 69	279 ± 79	292 ± 75	212 ± 62	257 ± 97	
	(30.7 ± 11.8)	(33.2 ± 8.7)	(31.7 ± 9.9)	(32.8 ± 9.3)	(37.2 ± 10.5)	(39.0 ± 10.0)	(28.2 ± 8.3)	(34.1 ± 12.9)	
T30	191 ± 50	210 ± 34	175 ± 43	183 ± 57	225 ± 57	226 ± 39	212 ± 55	244 ± 55	
	(25.5 ± 6.6)	(28.0 ± 4.5)	(23.4 ± 5.7)	(24.4 ± 7.6)	(30.0 ± 7.7)	(30.1 ± 5.2)	(28.3 ± 7.3)	(32.6 ± 7.3)	
T60	196 ± 84	205 ± 43	215 ± 72	214 ± 84	252 ± 46	222 ± 42	208 ± 27	251 ± 68	
	(26.1 ± 11.1)	(27.2 ± 5.7)	(28.7 ± 9.6)	(28.5 ± 11.2)	(33.6 ± 6.1)	(29.5 ± 5.6)	(27.6 ± 3.6)	(33.5 ± 9.1)	
Time effect		p < 0.01				ns			
Interaction		Ns				ns			
Group effect		Ns				ns			
**PaCO**_**2**_**[mmHg (kPa)]**									
BL1	34 ± 6	34 ± 5	34 ± 7	36 ± 6	33 ± 6	31 ± 7	31 ± 7	27 ± 4	
	(4.6 ± 0.7)	(4.6 ± 0.7)	(4.5 ± 0.9)	(4.8 ± 0.8)	(4.4 ± 0.8)	(4.2 ± 0.9)	(4.1 ± 0.9)	(3.6 ± 0.6)	
BL2	50 ± 7	43 ± 6	45 ± 6	50 ± 11	40 ± 7	42 ± 5	43 ± 9	39 ± 11	
	(6.7 ± 0.9)	(5.7 ± 0.9)	(6.0 ± 0.8)	(6.7 ± 1.5)	(5.3 ± 1.0)	(5.6 ± 0.7)	(5.8 ± 1.2)	(5.2 ± 1.5)	
T30	59 ± 13	47 ± 18	55 ± 12	58 ± 12	45 ± 9	56 ± 12	51 ± 11	44 ± 12	
	(7.8 ± 1.8)	(6.3 ± 2.4)	(7.4 ± 1.6)	(7.8 ± 1.6)	(6.0 ± 1.2)	(7.5 ± 1.6)	(6.9 ± 1.5)	(5.8 ± 1.6)	
T60	55 ± 16	55 ± 16	55 ± 9	48 ± 13	46 ± 11	55 ± 13	53 ± 8	52 ± 7	
	(7.4 ± 2.0)	(7.3 ± 2.2)	(7.3 ± 1.15)	(6.4 ± 1.7)	(6.2 ± 1.5)	(7.3 ± 1.7)	(7.0 ± 1.1)	(7.0 ± 0.9)	
Time effect		p < 0.01				p < 0.001			
Interaction		Ns				ns			
Group effect		Ns				ns			
**pHa**									
BL1	7.43 ± 0.08	7.44 ± 0.05	7.46 ± 0.06	7.46 ± 0.06	7.43 ± 0.05	7.45 ± 0.05	7.45 ± 0.07	7.46 ± 0.06	
BL2	7.30 ± 0.04	7.36 ± 0.06	7.33 ± 0.04	7.30 ± 0.06	7.32 ± 0.04	7.31 ± 0.04	7.32 ± 0.04	7.30 ± 0.06	
T30	7.24 ± 0.07	7.29 ± 0.07	7.28 ± 0.08	7.25 ± 0.08	7.24 ± 0.03	7.19 ± 0.09	7.23 ± 0.06	7.25 ± 0.08	
T60	7.24 ± 0.10	7.26 ± 0.08	7.28 ± 0.05	7.27 ± 011	7.25 ± 0.04	7.16 ± 0.10	7.20 ± 0.07	7.25 ± 0.07	
Time effect		p < 0.001				p < 0.001			
Interaction		Ns				ns			
Group effect		Ns				p < 0.01			
**BE [mmol/L]**									
BL1	−1 ± 4	−1 ± 2	0 ± 3	2 ± 1	−3 ± 2	−2 ± 3	−4 ± 2	5 ± 1	
BL2	−1 ± 2	−3 ± 2	−2 ± 1	−3 ± 1	−6 ± 4	−5 ± 4	−4 ± 4	−5 ± 2	
T30	−3 ± 4	−5 ± 4	−1 ± 2	−2 ± 2	−8 ± 4	−6 ± 5	−6 ± 4	−6 ± 3	
T60	−4 ± 6	−3 ± 2	−1 ± 3	−5 ± 4	−7 ± 4	−9 ± 6	−7 ± 4	−4 ± 3	
Time effect		p < 0.05				ns			
Interaction		Ns				ns			
Group effect		Ns				ns			
**HCO**_**3**_**[mmol/L]**									
BL1	23.1 ± 2.7	23.6 ± 2.3	24.5 ± 2.3	26.0 ± 1.6	22.0 ± 2.6	22.0 ± 2.9	21.0 ± 2.4	19.5 ± 1.3	
BL2	25.3 ± 1.7	23.1 ± 1.5	24.2 ± 0.9	24.3 ± 2.3	20.7 ± 3.6	21.8 ± 3.3	22.6 ± 4.0	21.6 ± 1.7	
T30	24.4 ± 3.3	22.2 ± 5.2	26.2 ± 2.0	25.5 ± 2.1	20.0 ± 3.7	22.0 ± 4.5	22.2 ± 3.7	21.9 ± 1.8	
T60	23.9 ± 5.3	24.7 ± 2.9	26.0 ± 2.8	21.9 ± 4.0	20.8 ± 4.1	20.2 ± 5.0	24.8 ± 10.8	23.2 ± 2.3	
Time effect		Ns				ns			
Interaction		Ns				ns			
Group effect		Ns				ns			

All values are expressed as mean and standard deviation. Differences between and within groups (*group and time effects, BL2-T60 as well as their interactions*) were tested with general linear model statistics and adjusted for repeated measures according to the Bonferroni method for ARDSp and ARDSexp separately. Statistical significance was accepted at α = 0.05 ns = non-significant; CTRL = control; SALB = salbutamol; it = intratracheally; iv = intravenously; ARDSp = pulmonary acute respiratory distress syndrome; ARDSexp = extrapulmonary acute respiratory distress syndrome; BL1 = baseline 1; BL2 = baseline 2; T30 and T60 = 30 and 60 min after initiation of therapy; PaO_2_ = arterial O_2_ partial pressure; PaCO_2_ = partial pressure of arterial CO_2_; pHa = arterial pH; BE = base excess; HCO_3_ = bicarbonate level.

**Figure 4:**
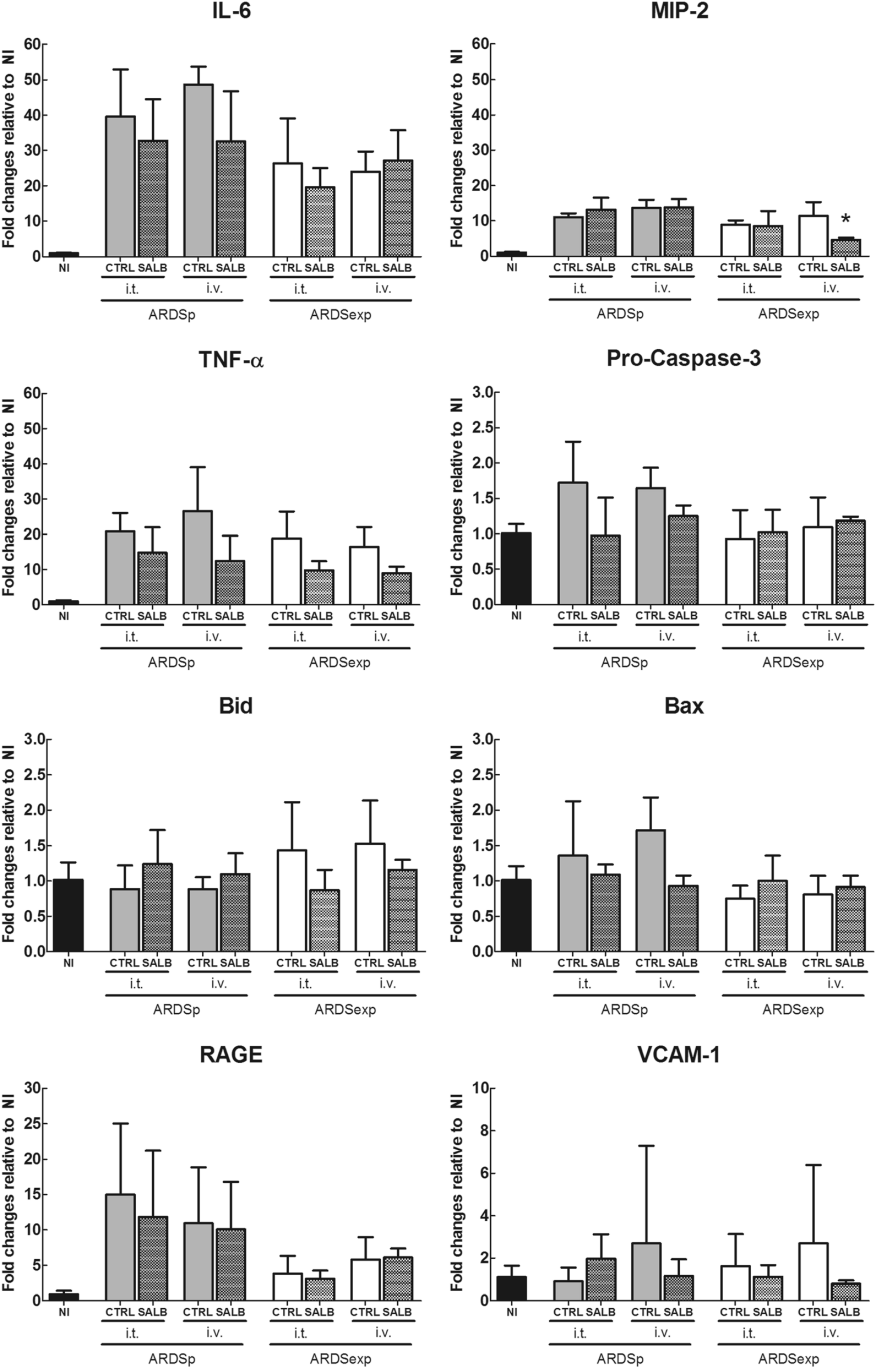
**Real-time polymerase chain reaction analysis of biological markers associated with inflammation [interleukin (IL)-6, macrophage inflammatory protein (MIP)-2, and tumor necrosis factor (TNF)-α], apoptosis [pro-caspase-3, BH3 interacting-domain death agonist (Bid), and Bcl-2-associated X protein (Bax)], and damage inflicted on alveolar type I epithelial cells [receptor for advanced glycation end-products (RAGE)] and endothelium [vascular cell adhesion molecule (VCAM)-1].** All values are expressed as mean and standard deviation. Differences between groups (SALB vs. CTRL; SALB-iv vs. SALB-it) were tested separately for ARDSp and ARDSexp using one-way ANOVA followed by Bonferroni correction. Statistical significance was accepted at α = 0.05. * = p < 0.05 vs. CTRL; NI = non-injured control; CTRL = control; SALB = salbutamol; i.t. = intratracheally; i.v. = intravenously; ARDSp = pulmonary acute respiratory distress syndrome; ARDSexp = extrapulmonary acute respiratory distress syndrome.

The W/D ratio and DAD score were not affected by salbutamol in any of the ARDS groups, regardless of the route of administration (Additional file 2: Figure S1 and Table 2, respectively). As shown in Figure 4, the gene expression of markers of inflammation, apoptosis, and endothelial and epithelial damage was not affected by intratracheal or intravenous administration of salbutamol in any of the ARDS groups.

**Table 2:** Diffuse alveolar damage (DAD) score

Variable	**ARDSp**				**ARDSexp**				
	**CTRL-it**	**SALB-it**	**CTRL-iv**	**SALB-iv**	**CTRL-it**	**SALB-it**	**CTRL-iv**	**SALB-iv**	
Cumulative (0–64)	11 [8–12]	9 [7–16]	11 [ 8–13]	13 [8–20]	8 [5–11]	8 [5–12]	8 [6–13]	9 [5–13]	ns
Edema (0–16)	1 [0–3]	1 [0–6]	2 [1–4]	2 [0–2]	0 [0–1]	1 [0–1]	1 [0–3]	1 [0–4]	ns
Hemorrhage (1–16)	2 [0–6]	2 [1–2]	1 [1–3]	3 [1–5]	2 [1–4]	2 [1–4]	1 [1–4]	2 [0–2]	ns
Inflammation (0–16)	4 [1–6]	2 [1–3]	2 [1–5]	4 [1–6]	2 [1–2]	2 [1–3]	1 [1–2]	1 [1–2]	ns
Overdistension (0–16)	4 [3–6]	4 [2–6]	4 [2–6]	4 [3–6]	3 [2–4]	4 [2–4]	4 [2–7]	3 [2–6]	ns

Values are expressed as median and interquartile range. Statistical analysis was performed using a mixed linear model with Tukey-Kramer adjustment for multiple comparisons. Statistical significance was accepted at α = 0.05. ns = non-significant; CTRL = control; SALB = salbutamol; it = intratracheally; iv = intravenously; ARDSp = pulmonary acute respiratory distress syndrome; ARDSexp = extrapulmonary acute respiratory distress syndrome.

## Discussion

The main findings of this study were that: 1) the gene expression of ENaC-α, AQP-1, AQP-3, and Na-K-ATPase-α was reduced in mild ARDSp, but not ARDSexp, 2) in mild ARDSp, intravenous salbutamol increased the gene expression of ENaC-α, AQP-1, AQP-3, and Na-K-ATPase-α, while intratracheal salbutamol increased the gene expression of AQP-1 and Na-K-ATPase-α, 3) in mild ARDSexp, the gene expression of ion channels and AQP was not influenced by salbutamol, regardless of the route of administration, and 4) morphofunctional variables and edema formation were not modulated by salbutamol, regardless of the ARDS model and route of drug administration.

To our knowledge, this is the first systematic investigation of the potential beneficial effects of salbutamol, administered by the intravenous and intratracheal routes, on lung morphology and function, inflammation, alveolar epithelial and endothelial cell damage, apoptosis, as well as on ion channels involved in the resolution of pulmonary edema in ARDSp and ARDSexp. In both models, the same agent, LPS, was used to induce ARDS. LPS challenges typically result in considerable tissue injury, characterized by neutrophil accumulation in the alveolar and interstitial spaces, alveolar wall thickening, and accumulation of proteinaceous edema in the alveolar space, which represent some of the hallmarks of experimental ARDS [22]. Salbutamol doses administered i.v. or i.t. were titrated based on HR, which is a clinically measurable target closely related to plasma drug concentration [23]. Salbutamol was chosen due to the following reasons: 1) it is a β2-agonist that has been previously used in clinical investigations of ARDS [14,15]; 2) it is available for both intravenous and intratracheal administration in the clinical setting; and 3) it can be easily titrated owing to its relatively short half-life.

It is worth noting that lung injury was associated with a decrease in the gene expression of ENaC-α, AQP-1, AQP-3, and Na-K-ATPase-α, as compared to non-injured animals, in ARDSp, but not in ARDSexp. In mild ARDSp, salbutamol increased the gene expression of ENaC-α, AQP-1, AQP-3, and Na-K-ATPase-α mainly through the intravenous route. Similar results with β2-agonists have been observed in different experimental studies [24-26]. The activation of such pathways may increase lung edema clearance, since in ARDSp the lung epithelium is the first structure to be damaged, with alveolar flooding and areas of consolidation [27]. However, we could not detect a reduction in pulmonary edema in our animals, as suggested by the measurement of W/D ratio. The gene expression associated with the mechanisms involved in edema clearance was enhanced, and it has been shown that channels translocation to membrane may occur within minutes [28,29] through a non-genomic, cyclic adenosine monophosphate (cAMP)-dependent pathway. On the other hand, the gene transcription could be mediated via the cAMP response binding element as demonstrated by the progression of lung inflammation [30,31]. To our knowledge, to date, this mechanism has not been investigated for fluid channels in the lung. However, these potential pathways were not translated into functional data of lung edema, as observed in Additional file 2: Figure S1. Allied to this, the time for building the respective proteins and structures was too short (1 hour), precluding the observation of an effect on edema clearance. Conversely, in mild ARDSexp, the first structure to be damaged is the endothelium, with subsequent increase in vascular permeability, microvascular congestion, and interstitial edema, but with relative sparing of the intra-alveolar spaces [27].

In ARDSexp, salbutamol did not affect the gene expression of ion channels and AQP, regardless of the route of administration. There are different possible explanations for the lack of effect of salbutamol on the gene expression of ion channels and AQP in ARDSexp: 1) the interstitial edema produces a barrier-like effect, thus hindering the diffusion of the intravenous β2-agonist into the alveolar epithelial layer; 2) impaired delivery of intravenous salbutamol to areas of microvascular congestion; 3) collapse of small airways and alveolar units, preventing the intratracheal drug from reaching the alveolar epithelium; and 4) almost normal gene expression, without room for improvement.

It has been suggested that β-agonists decrease the release of pro-inflammatory mediators [32], reduce cell apoptosis [33], and improve alveolar epithelial and endothelial repair in experimental lung injury [34,35]. Furthermore, it has been shown that β-agonists differentially affect ENaC channels by promoting the upregulation of selective and non-selective channels and that the route of drug delivery influences its effectiveness [36]. In the current study, the effect of salbutamol depended on the etiology of acute respiratory distress syndrome but not on the route of administration. In addition, salbutamol had no effect on inflammation, apoptosis or epithelial/endothelial repair, regardless of the etiology of ARDS and route of drug administration. There are different explanations for this discrepancy. First, it is possible that salbutamol doses required for inhibition of inflammation and enhancement of alveolar epithelial and endothelial repair are different from those required for gene regulation of ion channels and AQP. Second, since β2-agonists seem to have anti-inflammatory properties mainly in severe damage and inflammation [32], even promoting inflammation in the absence of pro-inflammatory stimuli [37], our animals were less likely to benefit from the anti-inflammatory effects of salbutamol, as they presented a mild form of acute lung injury. Third, we cannot refrain from mentioning that our experimental models of ARDS were less responsive than expected to the anti-inflammatory effects of β2-agonists, although such response has been shown not to be homogeneous across different models of lung injury [11]. Fourth, we cannot rule out the fact that anti-inflammatory, anti-apoptotic and alveolar epithelial/endothelial repair effects are time-dependent.

In both ARDS models, gas exchange and respiratory system mechanics were not affected by salbutamol, regardless of the route of administration. Inhaled salbutamol has been shown to reduce R_rs_ in patients with ARDS [38,39]. A possible explanation for the absence of beneficial effects on pulmonary function is the use of a relatively low PEEP level (3 cmH_2_O), which may have not been sufficient to keep distal airways and/or alveoli open. Thus, a possible beneficial effect of the β2-agonist on airway smooth muscle tone and gas exchange may have been overwhelmed by the effects on lung structures obtained with the ventilator settings. This hypothesis is supported by the fact that respiratory mechanics, and also oxygenation, worsened in all groups in a time-dependent manner. Because we did not assess the individual contributions of different components of R_rs_, we were not able to detect a possible effect on central airway resistance, which has been previously described [38]. It is worth noting that R_rs_ deteriorated after intratracheal administration of salbutamol in ARDSp. This finding is possibly related to the fact that the β2-agonist was administered as an intratracheal bolus, which may lead to partial mechanical obstruction of the airways. This hypothesis is supported by the observation that instillation of saline 0.9% i.t. produced a similar increase in R_rs_ in ARDSp.

### Possible clinical implications

The present study provides some mechanistic insights into the lack of positive effects of β2-agonists on clinical outcome in ARDS. Even though salbutamol upregulated the gene expression of ion channels and AQP in mild ARDSp, it did not enhance edema resolution. Furthermore, salbutamol did not upregulate the expression of those genes in ARDSexp, which showed a nearly normal expression. Thus, in the absence of positive effects, the side effects of β2-agonists may stand out.

### Limitations

Our study has several limitations. First, the models of mild ARDSp and ARDSexp used in the current study might not fully reproduce the complex features of clinical ARDS. Second, different salbutamol doses may result in different effects; thus, further studies are warranted to better address this issue. Third, mechanical ventilation with low tidal volumes led to hypercapnic acidosis, possibly interfering with anti-inflammatory effects [40]. Fourth, the 1-hour observation period was relatively short, precluding extrapolation of our findings to longer observation periods. In this context, even though the 1-hour period is sufficient to produce changes in gene expression, it may be too short to translate into protein synthesis.

## Conclusion

Salbutamol administration increased the expression of alveolar epithelial ion channels and AQP in mild ARDSp, but not ARDSexp, with no effect on lung morphology and function, inflammatory mediators, or edema formation. These results may contribute to explain the negative effects of β2-agonists on clinical outcome in ARDS.

### Key messages

•Gene expression of alveolar epithelial ion channels and aquaporin differed according to the etiology of ARDS.

•In mild ARDSp, both intratracheal and intravenous salbutamol increased the gene expression of alveolar epithelial ion channels and aquaporin.

•In mild ARDSexp, the gene expression of alveolar epithelial ion channels and aquaporin was not influenced by salbutamol.

•Salbutamol did not affect morphofunctional variables or edema formation, regardless of the ARDS model and route of drug administration.

## Abbreviations

AQP: Aquaporin; ARDS: Acute respiratory distress syndrome; BL: Baseline; cAMP: cyclic adenosine monophosphate; CTRL: Control; DAD: Diffuse alveolar damage; ECG: Electrocardiogram; exp: Extrapulmonary; ENaC: Epithelial sodium channel; Ers: Elastance of the respiratory system; FIO2: Fraction of inspired oxygen; HR: Heart rate; I:E: Inspiratory-to-expiratory ratio; i.p.: Intraperitoneally; i.t.: Intratracheally; i.v.: Intravenously; LPS: Lipopolysaccharide; MAP: Mean arterial pressure; p: Pulmonary; PaCO2: Partial pressure of arterial carbon dioxide; PaO2: Arterial oxygen partial pressure; Paw: Airway pressure; Pawmean: Mean airway pressure; Pawpeak: Peak airway pressure; PEEP: Positive end-expiratory pressure; pHa: Arterial pH; RR: Respiratory rate; Rrs: Resistance of the respiratory system; SALB: Salbutamol; T30 and T60: Measurement points at 30 min and 60 min after initiation of therapy; VT: Tidal volume; W/D ratio: Wet-to-dry ratio.

## Competing interests

The authors have no competing interests to declare.

This study was financially supported by a personnel exchange grant (PROBRAL) of the Coordination for the Improvement of Higher Education Personnel (CAPES), Brasília, Brazil, and the German Academic Exchange Service (DAAD), Bonn, Germany, as well as educational grants of the Carlos Chagas Filho Rio de Janeiro State Research Foundation (FAPERJ), Rio de Janeiro, Brazil, and the Brazilian Council for Scientific and Technological Development (CNPq), Brasília, Brazil.

## Authors’ contributions

CPU participated in the design of the study, carried out the experiments, performed data analyses and drafted the manuscript; PLS contributed to the study design, carried out the experiments, performed data analyses, and wrote the manuscript; DO carried out the molecular biology analyses and contributed to the manuscript; RSS and PJM provided expert assistance during experiments, analyzed mechanical data, and helped draft the manuscript; PMS and TK contributed to the study design and manuscript; MK performed the histological analyses and helped draft the manuscript; BW performed the statistical analyses and helped draft the manuscript; MMM contributed to study design and carried out the molecular biology analyses; PP contributed to the study design, supervised the entire project and helped write the manuscript; MGA and PRMR: contributed to the study design, supervised the experimental work and statistical analysis, wrote the manuscript, supervised the entire project. All authors read and approved the final manuscript.

## Supplementary Material

Additional file 1 This file gives detailed information about extended material and methods used including one table (Table S1 - Polymerase chain reaction (PCR) primers used in the study). Click here for file

Additional file 2 This file contains additional results covering two tables (Table S2 - Body weight, anesthesia and fluid therapy and Table S3 - Ventilator parameters and respiratory mechanics) and one figure (Figure S1 - Wet-to-dry (W/D) ratio). Click here for file
