# Supplementary material for: The effects of salbutamol on epithelial ion channels depend on the etiology of acute respiratory distress syndrome but not the route of administration
Source: Respir Res. 2014 May 2;15(1):56. doi: 10.1186/1465-9921-15-56 (PMC4026154; doi:10.1186/1465-9921-15-56)
Supplement: Additional file 1 — This file gives detailed information about extended material and methods used including one table (Table S1 - Polymerase chain reaction (PCR) primers used in the study). [file 1465-9921-15-56-S1.docx]

**Additional File 1**

**The effects of salbutamol on epithelial ion channels depend on the etiology of acute respiratory distress syndrome but not the route of administration**

Christopher P. Uhlig^1,2*^

Pedro L. Silva^1*^

Débora S. Ornellas^1,3^

Raquel S. Santos^1^

Paulo J. Miranda^1^

Peter M. Spieth^2^

Thomas Kiss^2^

Michael Kasper^4^

Bärbel Wiedemann^5^

Thea Koch^2^

Marcelo M. Morales^3^

Paolo Pelosi^6^

Marcelo Gama de Abreu^2^

Patricia R. M. Rocco^1^

*Dr. Uhlig and Dr. Silva contributed equally to this work.

^1^Laboratory of Pulmonary Investigation, Carlos Chagas Filho Biophysics Institute, Federal University of Rio de Janeiro, Av. Carlos Chagas Filho s/n, Bloco G-014, Ilha do Fundão, 21941-902, Rio de Janeiro, RJ, Brazil.

^2^Department of Anesthesiology and Intensive Care Therapy, Pulmonary Engineering Group, University Hospital Dresden, Technische Universität Dresden, Fetscherstr. 74, 01307, Dresden, Germany.

^3^Laboratory of Cellular and Molecular Physiology, Carlos Chagas Filho Biophysics Institute, Federal University of Rio de Janeiro, Av. Carlos Chagas Filho s/n, Bloco G2-048, Ilha do Fundão, 21941-902, Rio de Janeiro, RJ, Brazil.

^4^Institute of Anatomy, Faculty of Medicine, Technische Universität Dresden, Fetscherstr. 74, 01307, Dresden, Germany.

^5^Institute of Biometrics and Medical Informatics, Faculty of Medicine, Technische Universität Dresden, Fetscherstr. 74, 01307, Dresden, Germany.

^6^IRCCS AOU San Martino-IST, Department of Surgical Sciences and Integrated Diagnostics, University of Genoa, Largo Rosanna Benzi 8, 16132 Genoa, Italy.

*Correspondence and reprint requests to*:

Prof. Patricia R.M. Rocco, Laboratory of Pulmonary Investigation, Carlos Chagas Filho Biophysics Institute, Federal University of Rio de Janeiro, Rio de Janeiro, Brazil. Email: [prmrocco@biof.ufrj.br](mailto:prmrocco@biof.ufrj.br), Tel: +5521 25626530, Fax: +5521 22808193.

Prof. Marcelo Gama de Abreu, Pulmonary Engineering Group, Department of Anesthesiology and Intensive Care Medicine, Pulmonary Engineering Group, University Hospital Dresden, Technische Universität Dresden, Dresden, Germany. Email: [mgabreu@uniklinikum-dresden.de](mailto:mgabreu@uniklinikum-dresden.de), Tel: +493514584488, Fax: +493514584336.

**Material and Methods (extended)**

**Real-time polymerase chain reaction (PCR)/RNA quantification**

Central slices of the left lung were cut, collected in cryotubes, quick-frozen by immersion in liquid nitrogen, and stored at -80 °C. Total RNA was extracted using the SV Total RNA Isolation System (Promega, Fitchburg, WI, USA). RNA concentration was measured by spectrophotometry in Nanodrop® ND-1000 (NanoDrop products, Wilmington, DE, USA). First-strand cDNA was synthesized from total RNA using M-MLV Reverse Transcriptase Kit (Invitrogen, Carlsbad, CA, USA). Relative mRNA levels were measured with a SYBR green detection system (GoTaq® qPCR Master Mix; Promega, Madison, USA) in Eppendorf Mastercycler® ep realplex thermal cycler~~s~~ (Eppendorf, Hamburg, Germany). Samples were measured in triplicate. The PCR conditions for all primer sets were as follows: initial denaturation at 95 °C for 4 min followed by 40 amplification cycles, each consisting of 95 °C for 15 s, 60 °C for 30 s. Amplified PCR fragments were electrophoresed on 1.5 % agarose gels and stained with Gel Red (Biotium, Hayward, CA, USA). Relative gene expression was calculated as a ratio of average gene expression levels to the reference gene (cyclophilin A) and expressed as fold changes relative to non-injured and non-mechanically ventilated animals using the 2^-ΔΔCT^ method, where ΔCT = CT, reference gene - CT, target gene.

**Table S1** Polymerase chain reaction (PCR) primers used in the study

| **Oligonucleotides** | ***Sequence (5'-3')*** | ***Sequence (3'-5')*** |
| --- | --- | --- |
| Na-K-ATPase-α | GGC AGT GTT TCA GG CTA ACC AG | TTC TCC CTC ATC TCC ATC ACG G |
| ENaC-α | GGA CCA AGG AAC AAA TAG AAC AGC | TCA AGG AGA GGA GCA GAC ATC AG |
| AQP-1 | CTT ACC TCC AGG ACC CTT CC | TAG CTC ATC CAC ACG TGC TC |
| AQP-3 | AGC AGA TCT GAG TGG GCA GT | CTT GGG CTT AAG AGG GGA AC |
| TNF-α | CCC AGA CCC TCA CAC TCA G | CTC TGC TTG GTG GTT TGC T |
| IL-6 | CTC CGC AAG AGA CTT CCA G | CTC CTC TCC GGA CTT GTG A |
| MIP-2 | GCC TGG ATC GTA CCT GAT GT | GAG CTG GCC AAT GCA TAT CT |
| Pro-caspase-3 | GGC CGA CTT CCT GTA TGC | GCG CAA AGT GAC TGG ATG |
| Bid | GGC TAG CCG CTC CTT CTA TCA T | AGA TGC CTG GCA ATG TTG TGG A |
| Bax | ATC ATG GGC TGG ACA CTG GAC TT | TCC AGC CAC AAA GAT GGT CAC T |
| RAGE | TGA ACT CAC AGC CAA TGT CC | ACA ACT GTC CCT TTG CCA TC |
| VCAM-1 | TGC ACG GTC CCT AAT GTG TA | TGG ACC CAT TTC ACC TTT C |
| Cyclophilin A | TCC ACT TCG ATC TTG CCA CAG TCT | AGA CAC CAA TGG CTC CCA GTT CTT |

Na-K-ATPase-α = alpha subunit of the Na-K-ATPase; ENaC-α = alpha subunit of the epithelial sodium channel; AQP-1 = aquaporin 1; AQP-3 = aquaporin 3; TNF-α = tumor necrosis factor alpha; IL-6 = interleukin 6; MIP-2 = macrophage inflammatory protein-2; Bid = BH3 interacting-domain death agonist; Bax = Bcl-2-associated X protein; RAGE = receptor for advanced glycation end-products; VCAM-1 = vascular cell adhesion molecule-1.
