# Supplementary material for: The effects of salbutamol on epithelial ion channels depend on the etiology of acute respiratory distress syndrome but not the route of administration
Source: Respir Res. 2014 May 2;15(1):56. doi: 10.1186/1465-9921-15-56 (PMC4026154; doi:10.1186/1465-9921-15-56)
Supplement: Additional file 2 — This file contains additional results covering two tables (Table S2 - Body weight, anesthesia and fluid therapy and Table S3 - Ventilator parameters and respiratory mechanics) and one figure (Figure S1 - Wet-to-dry (W/D) ratio). [file 1465-9921-15-56-S2.docx]

**Additional File 2**

**The effects of salbutamol on epithelial ion channels depend on the etiology of acute respiratory distress syndrome but not the route of administration**

Christopher P. Uhlig^1,2*^

Pedro L. Silva^1*^

Débora S. Ornellas^1,3^

Raquel S. Santos^1^

Paulo J. Miranda^1^

Peter M. Spieth^2^

Thomas Kiss^2^

Michael Kasper^4^

Bärbel Wiedemann^5^

Thea Koch^2^

Marcelo M. Morales^3^

Paolo Pelosi^6^

Marcelo Gama de Abreu^2^

Patricia R. M. Rocco^1^

*Dr. Uhlig and Dr. Silva contributed equally to this work.

^1^Laboratory of Pulmonary Investigation, Carlos Chagas Filho Biophysics Institute, Federal University of Rio de Janeiro, Av. Carlos Chagas Filho s/n, Bloco G-014, Ilha do Fundão, 21941-902, Rio de Janeiro, RJ, Brazil.

^2^Department of Anesthesiology and Intensive Care Therapy, Pulmonary Engineering Group, University Hospital Dresden, Technische Universität Dresden, Fetscherstr. 74, 01307, Dresden, Germany.

^3^Laboratory of Cellular and Molecular Physiology, Carlos Chagas Filho Biophysics Institute, Federal University of Rio de Janeiro, Av. Carlos Chagas Filho s/n, Bloco G2-048, Ilha do Fundão, 21941-902, Rio de Janeiro, RJ, Brazil.

^4^Institute of Anatomy, Faculty of Medicine, Technische Universität Dresden, Fetscherstr. 74, 01307, Dresden, Germany.

^5^Institute of Biometrics and Medical Informatics, Faculty of Medicine, Technische Universität Dresden, Fetscherstr. 74, 01307, Dresden, Germany.

^6^IRCCS AOU San Martino-IST, Department of Surgical Sciences and Integrated Diagnostics, University of Genoa, Largo Rosanna Benzi 8, 16132 Genoa, Italy.

*Correspondence and reprint requests to*:

Prof. Patricia R.M. Rocco, Laboratory of Pulmonary Investigation, Carlos Chagas Filho Biophysics Institute, Federal University of Rio de Janeiro, Rio de Janeiro, Brazil. Email: [prmrocco@biof.ufrj.br](mailto:prmrocco@biof.ufrj.br), Tel: +5521 25626530, Fax: +5521 22808193.

Prof. Marcelo Gama de Abreu, Pulmonary Engineering Group, Department of Anesthesiology and Intensive Care Medicine, Pulmonary Engineering Group, University Hospital Dresden, Technische Universität Dresden, Dresden, Germany. Email: [mgabreu@uniklinikum-dresden.de](mailto:mgabreu@uniklinikum-dresden.de), Tel: +493514584488, Fax: +493514584336.

**Additional Results**


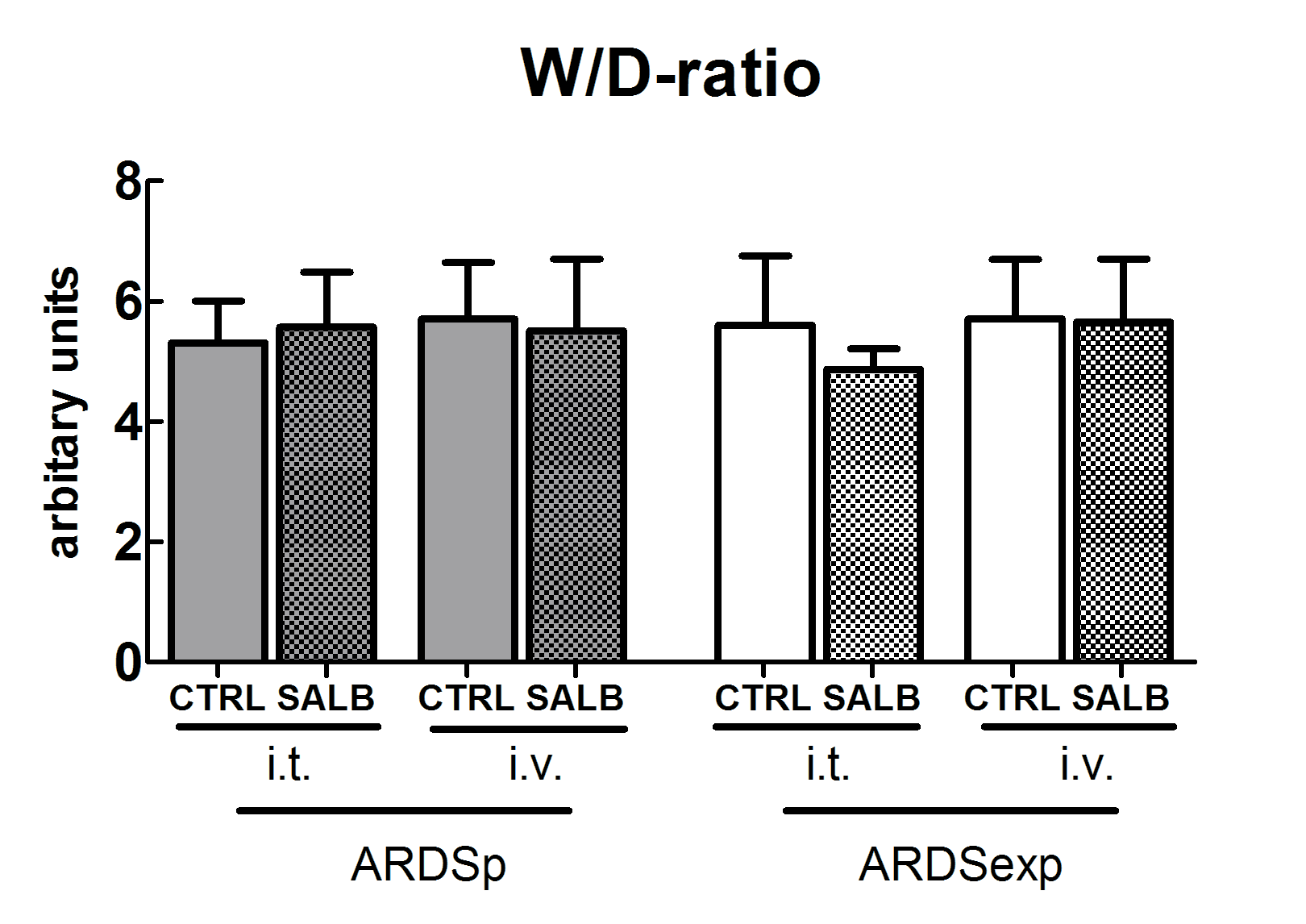


Figure S1: Wet-to-dry (W/D) ratio. All values are expressed as mean and standard deviation. Differences between groups (SALB vs. CTRL; SALB-iv vs. SALB-it) were tested separately for ARDSp and ARDSexp using one-way ANOVA followed by Bonferroni correction. Statistical significance was accepted at α = 0.05. CTRL = control; SALB = salbutamol; i.t. = intratracheally; i.v. = intravenously; ARDSp = pulmonary acute respiratory distress syndrome; ARDSexp = extrapulmonary acute respiratory distress syndrome.

**Table S2** Body weight, anesthesia and fluid therapy

| **Variable** | **ARDSp** | | | | **ARDSexp** | | | |  |
| --- | --- | --- | --- | --- | --- | --- | --- | --- | --- |
|  | CTRL-it | SALB-it | CTRL-iv | SALB-iv | CTRL-it | SALB-it | CTRL-iv | SALB-iv |  |
|  |  |  |  |  |  |  |  |  |  |
| **Body weight [kg]** | 0.300 ± 0.027 | 0.299 ± 0.029 | 0.290 ± 0.032 | 0.301 ± 0.035 | 0.294 ± 0.027 | 0.298 ± 0.036 | 0.295 ± 0.029 | 0.295 ± 0.015 | ns |
|  |  |  |  |  |  |  |  |  |  |
| **Midazolam [mg]** | 1.08 ± 0.24 | 1.12 ± 0.18 | 1.06 ± 0.26 | 1.05 ± 0.10 | 1.04 ± 0.20 | 1.05 ± 0.12 | 0.91 ± 0.29 | 0.98 ± 0.13 | ns |
|  |  |  |  |  |  |  |  |  |  |
| **Ketamine [mg]** | 54.1 ± 12.3 | 56.4 ± 9.5 | 53.5 ± 13.3 | 52.5 ± 5.0 | 52.3 ± 9.9 | 55.5 ± 8.7 | 45.8 ± 14.7 | 49.4 ± 6.8 | ns |
|  |  |  |  |  |  |  |  |  |  |
| **Crystalloid [mL]** | 4.3 ± 0.7 | 4.1 ± 0.4 | 4.3 ± 0.3 | 4.1 ± 0.5 | 4.3 ± 0.6 | 4.2 ± 1.0 | 4.5 ± 0.9 | 4.3 ± 4.5 | ns |
|  |  |  |  |  |  |  |  |  |  |
| **Colloid [mL]** | 1.1 ± 0.2 | 1.2 ± 0.3 | 1.6 ± 0.9 | 1.6 ± 0.7 | 1.4 ± 0.6 | 1.6 ± 0.6 | 1.1 ± 0.2 | 1.6 ± 0.7 | ns |
|  |  |  |  |  |  |  |  |  |  |

All values are expressed as mean and standard deviation. Differences between groups were tested separately for ARDSp and ARDSexp using one-way ANOVA followed by Bonferroni correction. Statistical significance was accepted at α = 0.05. ns = non-significant; CTRL = control; SALB = salbutamol; it = intratracheally; iv = intravenously; ARDSp = pulmonary acute respiratory distress syndrome; ARDSexp = extrapulmonary acute respiratory distress syndrome.

**Table S3** Ventilator parameters and respiratory mechanics

| Variable | | | **ARDSp** | | | | **ARDSexp** | | | |
| --- | --- | --- | --- | --- | --- | --- | --- | --- | --- | --- |
|  |  |  | CTRL-it | SALB-it | CTRL-iv | SALB-iv | CTRL-it | SALB-it | CTRL-iv | SALB-iv |
| **V_T_ [mL]** |  | |  |  |  |  |  |  |  |  |
|  | BL2 | | 6.02 ± 0.32 | 6.01 ± 0.45 | 6.01 ± 0.43 | 6.99 ± 0.46 | 6.08 ± 0.45 | 6.13 ± 0.33 | 5.94 ± 0.25 | 5.78 ± 0.32 |
|  | T30 | | 5.86 ± 0.28 | 6.04 ± 0.85 | 6.10 ± 0.52 | 6.27 ± 0.48 | 6.10 ± 0.35 | 6.10 ± 0.50 | 5.96 ± 0.62 | 6.02 ± 0.32 |
|  | T60 | | 6.19 ± 0.38 | 5.68 ± 0.37 | 6.60 ± 0.63 | 6.11 ±0.40 | 5.99 ± 0.51 | 6.31 ± 0.41 | 5.89 ± 0.81 | 6.02 ± 0.46 |
|  | Time effect | | Ns | | | | ns | | | |
|  | Interaction | | p<0.05 | | | | ns | | | |
|  | Group effect | | Ns | | | | ns | | | |
| **RR [1/min]** |  | |  |  |  |  |  |  |  |  |
|  | BL2 | | 79 ± 1 | 79 ± 1 | 79 ± 1 | 79 ± 1 | 79 ± 0 | 79 ± 1 | 79 ± 1 | 79 ± 1 |
|  | T30 | | 79 ± 1 | 80 ± 0 | 79 ± 1 | 79 ± 1 | 79 ± 1 | 80 ± 0 | 79 ± 1 | 79 ± 2 |
|  | T60 | | 79 ± 1 | 79 ± 1 | 79 ± 1 | 79 ± 1 | 79 ± 1 | 79 ± 1 | 79 ± 1 | 79 ± 1 |
|  | Time effect | | Ns | | | | ns | | | |
|  | Interaction | | Ns | | | | ns | | | |
|  | Group effect | | Ns | | | | ns | | | |
| **MV [L/min]** |  | |  |  |  |  |  |  |  |  |
|  | BL2 | | 0.143 ± 0.011 | 0.142 ± 0.019 | 0.143 ± 0.016 | 0.147 ± 0.017 | 0.139 ± 0.012 | 0.136 ± 0.010 | 0.136 ± 0.019 | 0.141 ± 0.010 |
|  | T30 | | 0.144 ± 0.022 | 0.149 ± 0.015 | 0.140 ± 0.020 | 0.150 ± 0.020 | 0.136 ± 0.015 | 0.145 ± 0.021 | 0.137 ± 0.011 | 0.141 ± 0.014 |
|  | T60 | | 0.152 ± 0.028 | 0.138 ± 0.017 | 0.140 ± 0.012 | 0.152 ± 0.021 | 0.136 ± 0.016 | 0.147 ± 0.021 | 0.137 ± 0.017 | 0.143 ± 0.008 |
|  | Time effect | | Ns | | | | ns | | | |
|  | Interaction | | Ns | | | | ns | | | |
|  | Group effect | | Ns | | | | ns | | | |
| **Paw_peak_ [cmH_2_O]** | | |  |  |  |  |  |  |  |  |
|  | BL2 | | 11.8 ± 5.5 | 10.0 ± 5.1 | 12.0 ± 3.5 | 14.6 ± 2.3 | 13.1 ± 3.6 | 13.8 ± 3.1 | 10.3 ± 3.3 | 11.8 ± 3.6 |
|  | T30 | | 14.6 ± 4.5 | 15.0 ± 5.3 | 12.6 ± 3.3 | 13.0 ± 3.1 | 11.6 ± 3.7 | 17.8 ± 4.1 | 14.5 ± 3.4 | 13.1 ± 2.7 |
|  | T60 | | 15.7 ± 4.3 | 13.8 ± 5.4 | 14.9 ± 2.8 | 14.7 ± 1.9 | 13.0 ± 1.5 | 16.1 ± 3.8 | 13.8 ± 3.5 | 12.5 ± 2.2 |
|  | Time effect | | p<0.05 | | | | p<0.05 | | | |
|  | Interaction | | Ns | | | | ns | | | |
|  | Group effect | | Ns | | | | ns | | | |
| **Paw_mean_ [cmH_2_O]** | | |  |  |  |  |  |  |  |  |
|  | | BL2 | 7.1 ± 5.4 | 8.1 ± 2.7 | 7.9 ± 1.6 | 9.5 ± 1.7 | 8.1 ± 2.8 | 9.0 ± 2.8 | 5.9 ± 2.2 | 7.0 ± 3.0 |
|  | | T30 | 8.0 ± 4.0 | 9.1 ± 4.9 | 8.0 ± 2.3 | 7.5 ± 2.3 | 7.1 ± 2.5 | 11.6 ± 4.0 | 9.1 ± 3.0 | 7.6 ± 2.2 |
|  | | T60 | 9.2 ± 5.0 | 8.0 ± 5.1 | 9.3 ± 2.3 | 8.4 ± 2.0 | 7.2 ± 1.5 | 9.8 ± 3.7 | 8.1 ± 3.2 | 6.8 ± 2.2 |
|  | | Time effect | Ns | | | | ns | | | |
|  | | Interaction | Ns | | | | ns | | | |
|  | | Group effect | Ns | | | | ns | | | |
| **E_rs_ [mL/cmH_2_O]** | | |  |  |  |  |  |  |  |  |
|  | BL2 | | 4.44 ± 0.88 | 3.56 ± 1.55 | 4.03 ± 1.09 | 4.62 ± 1.44 | 4.78 ± 0.91 | 4.53 ± 1.38 | 4.58 ± 1.25 | 4.03 ± 0.59 |
|  | T30 | | 5.48 ± 0.65 | 5.26 ± 1.25 | 4.83 ± 0.83 | 5.22 ± 1.76 | 5.19 ± 1.11 | 6.31 ± 1.81 | 6.13 ± 0.83 | 5.69 ± 1.63 |
|  | T60 | | 5.94 ± 1.27 | 5.73 ± 0.83 | 5.84 ± 1.13 | 6.48 ± 1.75 | 6.37 ± 1.28 | 6.56 ± 1.40 | 6.67 ± 0.83 | 5.93 ± 0.59 |
|  | Time effect | | p<0.001 | | | | p<0.001 | | | |
|  | Interaction | | Ns | | | | ns | | | |
|  | Group effect | | Ns | | | | ns | | | |
| **R_rs_ [cmH_2_O/L/s]** | | |  |  |  |  |  |  |  |  |
|  | BL2 | | 152.0 ± 21.9 | 181.0 ± 109.5 | 148.3 ± 66.5 | 151.3 ± 72.6 | 172.3 ± 50.8 | 194.0 ± 94.2 | 143.4 ± 57.8 | 161.8 ± 59.2 |
|  | T30 | | 177.3 ± 32.8 | 269.5 ± 109.1 | 163.3 ± 44.5 | 185.9 ± 122.9 | 205.3 ± 85.5 | 265.7 ± 139.1 | 262.6 ± 129.4 | 236.3 ± 92.8 |
|  | T60 | | 168.9 ± 58.8 | 265.2 ± 161.7 | 195.9 ± 74.2 | 236.3 ± 108.4 | 275.3 ± 168.1 | 226.7 ± 75.5 | 278.1 ± 168.9 | 252.4 ± 114.6 |
|  | Time effect | | p<0.05 | | | | p<0.001 | | | |
|  | Interaction | | Ns | | | | ns | | | |
|  | Group effect | | Ns | | | | ns | | | |

All values are expressed as mean and standard deviation. Differences between and within groups (*group and time effects, BL2-T60 as well as their interactions*) were tested with two-way ANOVA and adjusted for repeated measures according to the Bonferroni method for ARDSp and ARDSexp separately. Statistical significance was accepted at α = 0.05. ns = non-significant; CTRL = control; SALB = salbutamol; it = intratracheally; iv = intravenously; ARDSp = pulmonary acute respiratory distress syndrome; ARDSexp = extrapulmonary acute respiratory distress syndrome; BL2 = baseline 2; T30 and T60 = 30 and 60 min after initiation of therapy; V_T_ = tidal volume; RR = respiratory rate; MV = minute ventilation; Paw_peak_ = peak inspiratory airway pressure; Paw_mean_ = mean inspiratory airway pressure; E_rs_ = elastance of the respiratory system; R_rs_ = resistance of the respiratory system.
